# 785. Distribution and Associated Mortality in Carbapenem-Resistant Gram-Negative Bacilli in Japan: A Multicenter Study From Multi-Drug Resistant Organisms Clinical Research Network (MDRnet)

**DOI:** 10.1093/ofid/ofab466.982

**Published:** 2021-12-04

**Authors:** Sho Saito, Aki Sakurai, Kohei Uemura, Yasufumi Matsumara, Ryota Hase, Hideaki Kato, Masahiro Suzuki, Mari Kurokawa, Kota Sakamoto, Koh Shinohara, Kayoko Sano, Tetsuya Suzuki, Kayoko Hayakawa, David van Duin, Norio Ohmagari, Yohei Doi, Yohei Doi

**Affiliations:** 1 Disease Control and Prevention Center, National Center for Global Health and Medicine, Tokyo, Japan, Shinjukuku, Tokyo, Japan; 2 University of Texas Health Science Center, McGovern Medical School, Houston, TX; 3 The University of Tokyo, Bunkyo-ku, Tokyo, Japan; 4 Kyoto University Graduate School of Medicine, Kyoto, Kyoto, Japan; 5 Japanese Red Cross Narita Hospital/Kameda Medical Center, Narita, Chiba, Japan; 6 Yokohama City University Graduate School of Medicine, Yokohama, Kanagawa, Japan; 7 Fujita Health University School of Medicine, Toyoake, Aichi, Japan; 8 Okayama University Hospital, Kitaku, Okayama, Japan; 9 Yokohama City University Hospital, Kanazawa-ku, Kanagawa, Japan; 10 National Center for Global Health and Medicine, Shinjuku-ku, Tokyo, Japan; 11 National Center for Global Health and Medicine Hospital, Shinjuku, Tokyo, Japan; 12 University of North Carolina, Chapel Hill, North Carolina; 13 University of Pittsburgh, Pittsburgh, PA

## Abstract

**Background:**

Carbapenem-resistant gram-negative bacilli (CRGNB) are increasingly reported around the world as a cause of serious infections. However, the epidemiology and clinical course of patients with CRGNB in Japan is not well understood.

**Methods:**

We prospectively collected CR cases from 4/2019 to 9/2020 in Multi-Drug Resistant organisms clinical research network (MDRnet) consisting of 5 tertiary care facilities in Japan. We looked for all CRGNB, and all unique patients with CR *Enterobacterales*, CR nonfermenting gram-negative bacilli (NFGNB) and CR *Aeromonas* sp. isolation were included. Carbapenem resistance was tested by agar dilution method and defined based on the CLSI criteria for each species. Infections were determined by NHSN protocols.

**Results:**

In total, 156 patients (30 *Enterobacterales*, 119 NFGNB, 7 *Aeromonas* spp.) were included (11 *Enterobacter* spp., 11 *Klebsiella* spp., 86 *Pseudomonas aeruginosa*, 29 *Stenotrophomonas maltophilia,* 7 *Aeromonas* spp.). *Acinetobacter* sp. was not detected. Isolation sites were sputum (n = 12) and urine (n = 7) in *Enterobacterales*, sputum (n = 62) and blood (n = 18) in NFGNB, and blood (n = 6) in *Aeromonas* spp. The median age and male ratio of the patients were 68 years [IQR: 53-74] and 19 (63.3%) in *Enterobacterales*, 72 years [IQR: 60-79] and 70 (58.8%) in NFGNB and 78 years [IQR: 54-83] and 2 (28.6%) in *Aeromonas* spp. Ten (33.3%) patients with *Enterobacterales*, 55 (46.2%) patients with NFGNB, and 6 (85.7%) patients with *Aeromonas* spp. were infected cases. The others were considered as colonized. There were no patients with ICU stay or intubation in *Enterobacterales*, while 5 (4.2%) and 4 (3.4%) patients were in ICU and intubated in NFGNB, and 2 patients were in ICU and intubated in *Aeromonas* spp., respectively. All-cause 30-day mortality rates were 10% in *Enterobacterales*, 16.8 % in NFGNB and 28.6% in *Aeromonas* spp. In the infected patients, 3 patients (30%) with *Enterobacterales*, 12 patients (21.8%) with NFGNB and 1 patient (16.7%) with *Aeromonas* spp. died within 30 days after isolation.

Flow diagram outlining the characteristics of the patients and species in this study.

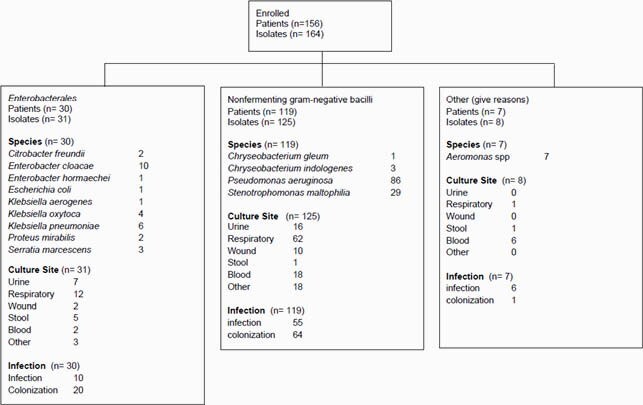

Kaplan-Meier survival curves of patients with carbapenem resistant Enterobacterales

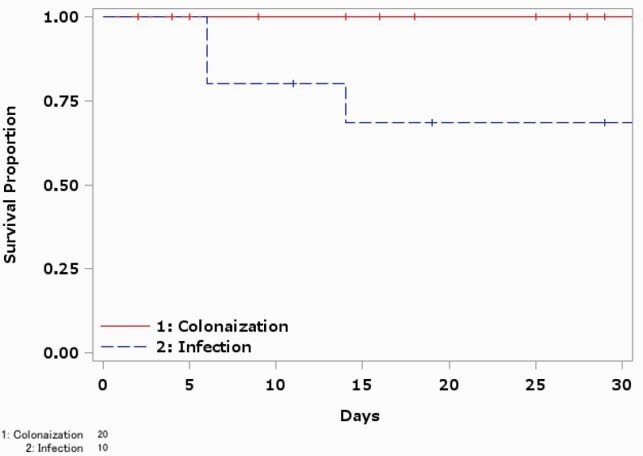

Kaplan-Meier survival curves of patients with carbapenem resistant nonfermenting gram-negative bacilli

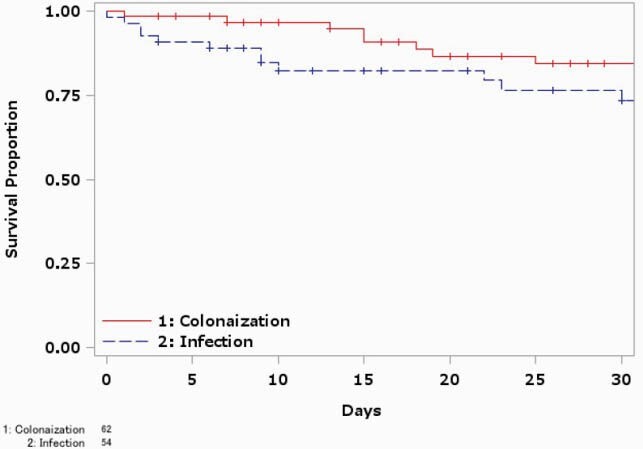

**Conclusion:**

Mortality rates were high in infected cases of CR *Enterobacterales*, CR NFGNB and CR *Aeromonas* spp. Carbapenem-resistant *Acinetobacter* spp. was not detected, which differed from the CR epidemiology in Europe, the United States, and other Asian countries.

**Disclosures:**

**Sho Saito, n/a**, **Shionogi** (Grant/Research Support) **David van Duin, MD, PhD**, **Entasis** (Advisor or Review Panel member)**genentech** (Advisor or Review Panel member)**Karius** (Advisor or Review Panel member)**Merck** (Grant/Research Support, Advisor or Review Panel member)**Pfizer** (Consultant, Advisor or Review Panel member)**Qpex** (Advisor or Review Panel member)**Shionogi** (Grant/Research Support, Scientific Research Study Investigator, Advisor or Review Panel member)**Utility** (Advisor or Review Panel member) **Yohei Doi, MD, PhD**, **AstraZeneca** (Speaker's Bureau)**bioMerieux** (Consultant)**FujiFilm** (Advisor or Review Panel member, Speaker's Bureau)**Gilead** (Consultant)**GSK** (Consultant)**Meiji** (Consultant)**MSD** (Consultant)**Shionogi** (Consultant) **Yohei Doi, MD, PhD**, Astellas (Individual(s) Involved: Self): Grant/Research Support; AstraZeneca (Individual(s) Involved: Self): Speakers' bureau; bioMerieux (Individual(s) Involved: Self): Consultant, Speakers' bureau; Chugai (Individual(s) Involved: Self): Consultant; Entasis (Individual(s) Involved: Self): Consultant; FujiFilm (Individual(s) Involved: Self): Advisor or Review Panel member; Gilead (Individual(s) Involved: Self): Consultant; GSK (Individual(s) Involved: Self): Consultant; Kanto Chemical (Individual(s) Involved: Self): Grant/Research Support; MSD (Individual(s) Involved: Self): Speaking Fee; Pfizer (Individual(s) Involved: Self): Grant/Research Support; Shionogi (Individual(s) Involved: Self): Grant/Research Support, Speakers' bureau; Teijin Healthcare (Individual(s) Involved: Self): Speakers' bureau; VenatoRx (Individual(s) Involved: Self): Consultant

